# Dysregulation of miR-637 serves as a diagnostic biomarker in patients with carotid artery stenosis and predicts the occurrence of the cerebral ischemic event

**DOI:** 10.1080/21655979.2021.1988369

**Published:** 2021-10-18

**Authors:** Ting Zhang, Ruijie Liu

**Affiliations:** a Department of Preventive Medicine, Dongying People’s Hospital, Dongying, China; b Department of Vertigo Division, Dongying People’s Hospital, Dongying, China

**Keywords:** Serum miR-637, diagnostic, carotid artery stenosis

## Abstract

The present research aims to explore the relationship between circulating microRNA and carotid artery stenosis (CAS). To evaluate the diagnostic significance of miR-637 in CAS patients and its potential predictive value for cerebral ischemia events through clinical studies. Quantitative reverse transcription-polymerase chain reaction (qRT-PCR) was used to verify the differences in serum miR-637 between enrolled 97 CAS patients and 90 healthy individuals. Logistic regression analysis of the correlation between the level of miR-637 and the degree of carotid artery stenosis. The receiver operating characteristic (ROC) curve evaluated the diagnostic significance of miR-637 in identifying CAS patients from healthy individuals. Kaplan-Meier survival and Cox regression were used to evaluate the potential predictive ability of serum miR-637 levels during follow-up for cerebral ischemia events. Serum miR-637 of CAS patients was significantly reduced which was a good indicator of severe carotid stenosis (*P* < 0.001). Reduced miR-637 can identify CAS patients from healthy individuals, demonstrating strong diagnostic capabilities. Furthermore, Kaplan-Meier analysis confirmed that the lower miR-637 levels in CAS, the more cerebral ischemia events (log-rank, *P* = 0.035), and the Multivariate Cox regressions confirmed that miR-637 was an independent predictor of CAS patients (HR = 0.073, 95%CI = 0.017–0.313, *P* < 0.001). We confirmed that serum miR-637 in CAS patients was significantly reduced. And reduced miR-637 was not only a potentially reliable biomarker for the diagnosis of CAS but also a useful indicator for predicting future cerebral ischemic events.

## Introduction

Stroke is the most common cause of long-term disability and the third leading cause of death after heart disease and cancer [1]. In China, there are 1.5 million new stroke patients every year, of which ischemic stroke accounts for about 80% [2]. Symptomatic carotid artery stenosis (CAS) is a key factor in cerebral ischemia events. When the degree of stenosis ≥50%, the incidence of cerebral ischemic events increases by 10%-15% [3]. Therefore, since CAS is a recognized risk factor for ischemic stroke [4], looking for biomarkers of CAS is of great significance for preventing the occurrence of ischemic events. Currently, digital subtraction angiography (DSA), computed tomography angiography (CTA), and magnetic resonance angiography (MRA) have been used to identify representative methods of CAS [5]. However, most CAS develops from asymptomatic simple intima-media thickening to symptomatic stenosis, which requires regular testing and is expensive [5], and the contrast agents used in both methods may pose a risk of adverse effects in patients [6]. Doppler ultrasound is a common aspect of CAS noninvasive testing despite its advantages of security, simplicity, and low cost. However, its detection results are easily affected by the technical level of operators, and it is not suitable for screening large populations. Therefore, finding a new method of CAS diagnosis and progress is of great significance to prevent the occurrence of cerebral ischemia events.

In recent years, microRNAs have received widespread attention for regulating their target mRNA in the pathological process of human diseases. For example, miR-146a [7], miR-214 [8], miR-638 [9], and miR-330-5p [10] were all involved in CAS, suggesting that miRNA dysregulation may have the potential regulatory effect on CAS. Besides, miRNAs can be used as disease biomarkers and therapeutic targets due to their stability and conservatism in blood. MiR-3352 [11], miR-451 [12], miR-599 [13] can be used as potential biomarkers of CAS. Among a variety of miRNAs, miR-637 is located on chromosome 19p13.3, and its effect on hepatoma [14], cholangiocarcinoma [15], gastric cancer [16], melanoma [17], and other diseases have been confirmed. Xu et al. recently demonstrated that serum miR-637 is a hallmark of decreased atherosclerosis and can predict the occurrence of cardiovascular events [18]. It has been reported that miR-637 is down-regulated and negatively correlated with serum procalcitonin in patients with ischemic stroke [19]. Abnormal regulation of miR-637 is also involved in the development of retinopathy in hypertensive patients [20]. Although miR-637 has been reported in vascular-related diseases such as atherosclerosis, ischemic stroke, and hypertension, its clinical significance in CAS has not been explored.

Considering the pathogenic role of abnormal miR-637 in the development of cardiovascular and cerebrovascular diseases, we hypothesized that circulating miR-637 may also be associated with CAS. And carry out clinical research to evaluate the diagnostic significance of miR-637 in CAS patients and its potential predictive value for cerebral ischemia events.

## Materials and methods

### Subject’s involvement

A total of 97 CAS patients volunteers (63.26 ± 7.29 years) and 90 age-matched healthy individuals (63.61 ± 7.39 years) were selected from Dongying People’s Hospital from June 2013 to November 2015 were included in this study. The current study was reviewed and approved by the Dongying People’s Hospital System Review Committee, and this study was carried out with the informed consent of patients. Color doppler ultrasonography, CTA, MRA, and DSA screening were performed before enrollment. And according to the previous definition of asymptomatic CAS [21], the inclusion criteria were patients with a degree of stenosis ≥ 50% on DSA and no recent transient ischemic attack, amaurosis, ipsilateral carotid artery ischemic stroke, and patients with diabetes and other CAS susceptible groups as well as those who had received antithrombotic treatment were excluded. According to previous studies [22], the range of moderate carotid stenosis is 60%-69%, and severe carotid stenosis is ≥ 70%. The healthy individuals were all from the health examination center, and there were no cardiovascular and cerebrovascular diseases, acute or chronic inflammation, and autoimmune diseases. The clinical data of all subjects were summarized in Table 1.

**Table 1.** Clinical data of the study population

Variables	All subjects (N = 187)		*P-*value
	Healthy individuals (n = 90)	CAS patients (n = 97)	
Gender (Male and female)	43/47	50/47	0.607
Age (years)	63.61 ± 7.39	63.26 ± 7.29	0.742
BMI (kg/m^2^)	23.12 ± 2.66	22.77 ± 2.88	0.388
FBG (mg/dL)	93.25 ± 17.38	96.90 ± 15.91	0.136
TC (mg/dL)	192.11 ± 4.75	191.78 ± 4.07	0.613
TG (mg/dL)	122.89 ± 12.89	123.48 ± 13.44	0.758
HDL (mg/dL)	49.38 ± 3.71	48.81 ± 3.77	0.306
LDL (mg/dL)	110.61 ± 5.65	112.44 ± 6.95	0.056
SBP (mm Hg)	126.46 ± 12.62	129.76 ± 13.34	0.066
DBP (mm Hg)	78.80 ± 11.41	81.73 ± 10.44	0.056
Degree of carotid artery stenosis	-	65.69 ± 11.40	-

Abbreviations: CAS, carotid stenosis; BMI, body mass index; FBG, fasting blood glucose; TC, total cholesterol; TG, triglycerides; HDL, high-density lipoprotein; LDL, low density lipoprotein; SBP, systolic blood pressure; DBP, diastolic blood pressure. Data are expressed as n or mean ± standard deviation.

### Serum sample collection

After 8 h of fasting, the peripheral venous blood of the upper extremity of the subjects was collected to determine blood lipids and serum glucose [23]. After standing at room temperature, centrifuge at 2000 rpm/min for 10 min. Collect the serum and store it at −80°C.

### Follow-up

Telephone followed up for 5 years. The follow-up endpoint was the occurrence of cerebral ischemic events, such as ipsilateral stroke, transient ischemic attack, amaurosis, or sudden death.

### qRT-PCR reaction

Trizol and miRNase Mini Kit were used to separate and purify total RNA from patients. Determine the concentration and quality of RNA by spectrophotometer. 500 ng RNA extracted was then reverse-transcribed into complementary DNA (cDNA) [24] by the miRcute Plus miRNA First-Strand cDNA Kit. The cDNA samples were then amplified on the 7900 HT Fast Real-Time PCR System (Life Technologies) using the miRcute Plus miRNA qPCR Kit (SYBR Green). U6 snRNA was used as internal reference, and the relative expression of miR-637 was calculated using by 2^−ΔΔCt^.

### Statistical analysis

The differences between groups were statistically analyzed by student’s t-test and one-way ANOVA followed by the post hoc Tukey’s test. ROC curve assessed the diagnostic significance of miR-637 in distinguishing CAS patients from healthy individuals. Kaplan-Meier and Cox regression test the predictive potential of miR-637 for cerebral ischemia events. All data were analyzed using SPSS 23.0 software and GraphPad Prism 6.0 software. *P* < 0.05 was considered statistically significant.

## Results

Due to the important role of miR-637 in other diseases, we recruited asymptomatic CAS and healthy individuals. the qRT-PCR reaction was used to compare the levels in the two groups of subjects, and its potential clinical diagnostic value and ability to predict cerebral ischemic events were further explored.

### Clinical characteristics of the subjects

Table 1 included the clinical characteristics of CAS patients (n = 97) and healthy individuals (n = 90). CAS patients (63.26 ± 7.29 years) including 50 males and 47 females. Healthy individuals (63.61 ± 7.39 years) were 43 male and 47 female. CAS’s patients’ age, body mass index (BMI), fasting blood glucose (FBG), triglycerides (TG), total cholesterol (TC), high-density lipoprotein (HDL), low-density lipoprotein (LDL), systolic blood pressure (SBP), and diastolic blood pressure (DBP) compared with healthy individuals, the difference was not statistically significant (*P* >0.05).

### Serum miR-637 was decreased in CAS patients significantly

**Figure 1.**
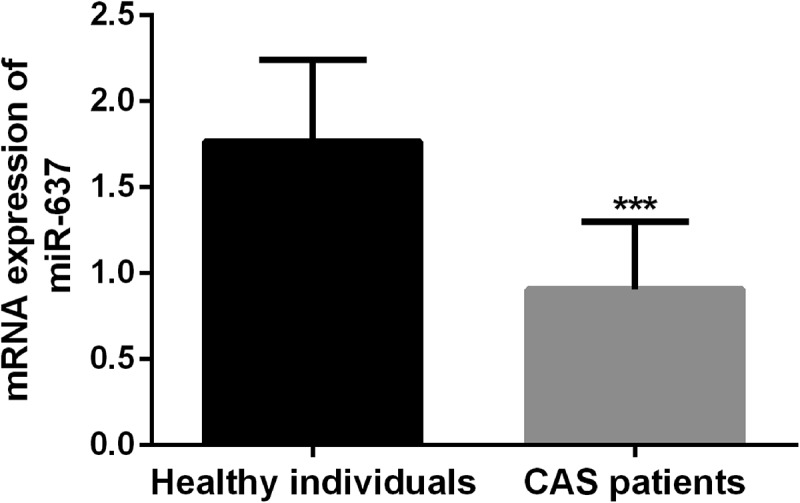
Serum miR-637 expression levels in healthy individuals and CAS patients were quantified by the qRT-PCR reaction. The expression level of serum miR-637 was nearly twofold lower in patients with CAS. *** *P* < 0.001, compared with healthy individuals

Next, the serum samples of CAS patients and healthy individuals were verified for miR-637 levels by qRT-PCR. Figure 1 shows that serum miR-637 levels in CAS patients were typically decreased (*P* < 0.05). The results suggest that the up-regulation of miR-637 may play a potential role in CAS.

### Serum miR-637 is a good indicator of high carotid artery stenosis

Subsequently, logistic regression was used to analyze the influencing factors of CAS degree in CAS patients. According to the degree of carotid artery stenosis, CAS patients were divided into severe stenosis group (n = 35) and moderate stenosis group (n = 62). Table 2 confirmed that, compared to other clinical indicators, the reduction of serum miR-637 was a good independent factor for severe carotid artery stenosis in CAS patients (OR = 0.050, 95% CI = 0.014–0.174, *P* < 0.001). The results suggest that serum miR-637 is a good indicator of severe carotid artery stenosis.

**Table 2.** Association of different variables with the occurrence of degree of carotid artery stenosis

Variables	OR	95% CI	*P* value
MiR-637	0.050	0.014–0.174	<0.001
Gender (Male and female)	0.755	0.237–2.404	0.634
Age (years)	0.875	0.297–2.578	0.809
BMI (kg/m^2^)	0.853	0.285–2.550	0.776
FBG (mg/dL)	0.373	0.118–1.172	0.091
TC (mg/dL)	0.809	0.280–2.336	0.695
TG (mg/dL)	0.625	0.197–1.981	0.424
HDL (mg/dL)	0.491	0.164–1.465	0.202
LDL (mg/dL)	1.741	0.560–5.411	0.338
SBP (mm Hg)	0.859	0.227–3.252	0.822
DBP (mm Hg)	0.741	0.233–2.360	0.612

Abbreviations: BMI, body mass index; FBG, fasting blood glucose; TC, total cholesterol; TG, triglycerides; HDL, high-density lipoprotein; LDL, low density lipoprotein; SBP, systolic blood pressure; DBP, diastolic blood pressure.

### Diagnostic role of serum miR-637 in CAS patients

**Figure 2.**
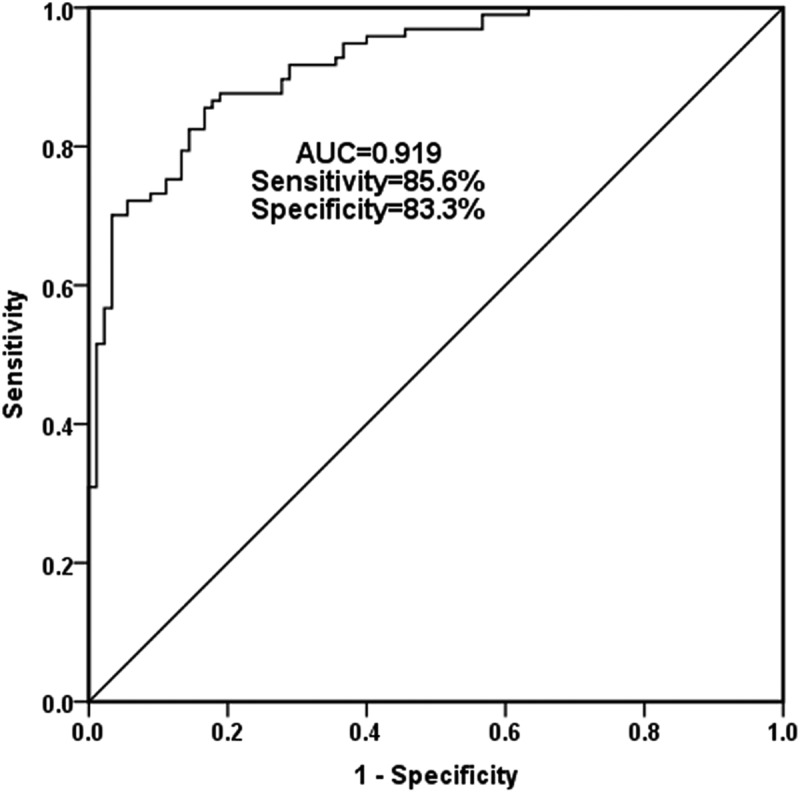
ROC curve showed the potential diagnostic capacity of serum miR-637 level in patients with CAS. The area under the curve (AUC) was 0.919. When the optimal cutoff point was 0.759, the sensitivity was 85.6%, and specificity was 83.3%

Given that miR-637 was an independent indicator of stenosis, this part of the study further evaluates the accuracy of serum miR-637 as a potential diagnostic biomarker to distinguish CAS patients from the healthy individual by ROC curve. Figure 2 confirmed that serum miR-637 has significant diagnostic ability in distinguishing CAS from healthy individuals, with an AUC of 0.919, and a best cutoff point of 0.759, indicating a sensitivity of 85.6%, and a specificity of 83.3%. Current research confirmed that serum miR-637 is a valuable diagnostic biomarker for CAS patients.

### Predictive value of serum miR-637 for the occurrence of the cerebral ischemic event

**Figure 3.**
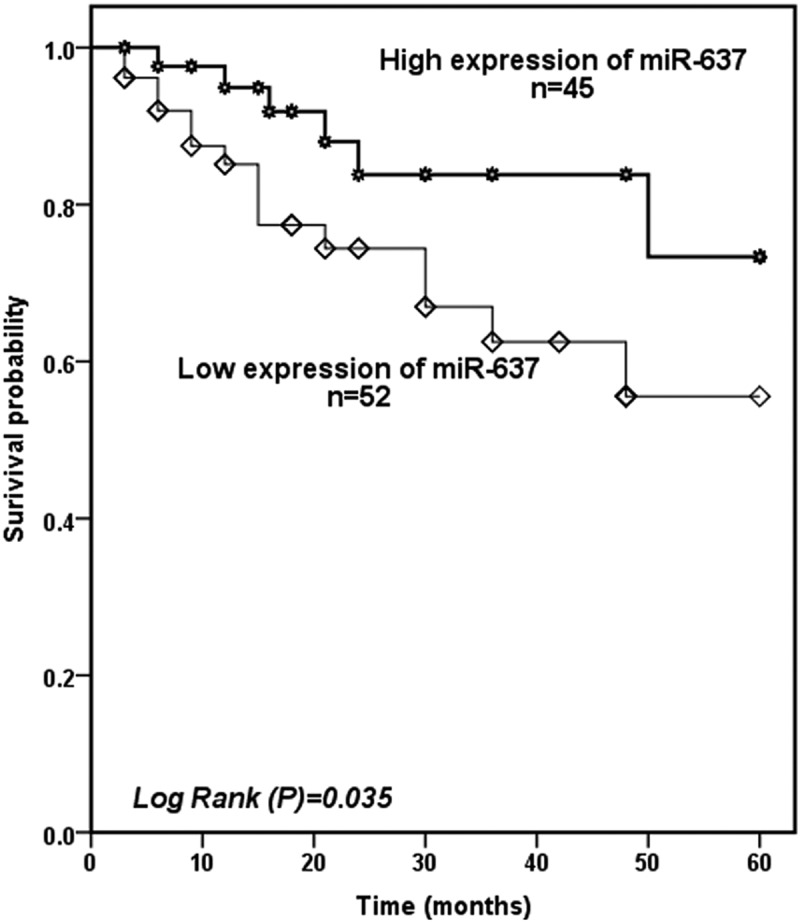
Kaplan-Meier curve was used to evaluate the predictive value of serum miR-637 for cerebral ischemia events. It was found that patients with lower miR-637 levels developed more ischemic events (log-rank *P* = 0.035)

In the final part of this research, we examine the predictive ability of serum miR-637 for cerebral ischemic events. To facilitate analysis, we divided patients into the high expression group (n = 45) and low expression group (n = 52) based on the average level of miR-637 (0.52 ± 0.22) of patients. During the 5 years follow-up, the Kaplan-Meier survival curve was drawn based on the patients’ cerebral ischemic events confirmed that patients with low miR-637 levels experienced more cerebral ischemic events (log-rank *P* = 0.035, Figure 3). Subsequently, multivariate Cox regression analysis confirmed serum miR-637 (HR = 0.073, 95%CI = 0.017–0.313, *P* < 0.001) and severe carotid artery stenosis (HR = 0.144, 95%CI = 0.045–0.463, *P* = 0.001) were high risk factor for cerebral ischemic events (Table 3).

**Table 3.** Multivariate Cox analysis of clinical characteristics in relation to overall survival

Characteristics	Multivariate analysis		
	HR	95% CI	*P*
MiR-637	0.073	0.017–0.313	<0.001
Gender (Male and female)	0.595	0.204–1.740	0.343
Age (years)	2.241	0.762–6.589	0.143
BMI (kg/m^2^)	0.464	0.161–1.333	0.154
FBG (mg/dL)	0.594	0.219–1.614	0.307
TC (mg/dL)	0.598	0.206–1.735	0.344
TG (mg/dL)	0.516	0.165–1.616	0.256
HDL (mg/dL)	0.517	0.176–1.518	0.230
LDL (mg/dL)	1.559	0.531–4.579	0.419
SBP (mm Hg)	0.511	0.138–1.892	0.315
DBP (mm Hg)	0.346	0.120–0.999	0.050
Degree of carotid artery stenosis	0.144	0.045–0.463	0.001

Abbreviations: BMI, body mass index; FBG, fasting blood glucose; TC, total cholesterol; TG, triglycerides; HDL, high-density lipoprotein; LDL, low density lipoprotein; SBP, systolic blood pressure; DBP, diastolic blood pressure.

## Discussion

CAS refers to the stenosis or contraction of the internal surface of the carotid artery and even the occlusion of the inner diameter of the artery. Prolonged stenosis can lead to chronic cerebral ischemia and hypoxia, which can lead to cerebral ischemic events such as ischemic stroke or transient ischemic accident [25,26]. Despite significant progress in prevention and treatment, stroke is still one of the leading causes of disability and death [27]. Thromboembolism is caused by 50% to 99% of internal carotid stenosis, which accounts for 10%-15% of all strokes [28]. Therefore, effective diagnosis and treatment of CAS contribute to the occurrence of cerebral ischemic events. Although DSA is currently the gold standard for the diagnostic of CAS at present, and it can describe the location, scope, and degree of lesions in detail, this kind of angiography is a traumatic detection method with the high cost and the possibility of complications such as cerebral vasospasm and cerebral embolism. In addition, Doppler ultrasound is currently the preferred noninvasive CAS detection method, which is simple, safe, and low-cost. However, it cannot detect intracranial CAS lesions, and the results are easily affected by the skill level of the operator [29]. Therefore, dynamic circulating blood and serum biomarkers have attracted extensive attention [7]. And because of their monitoring reagents, instruments are available in general laboratories and their experimental technology is mature, suitable for large-scale screening. In addition, circulating blood and serum samples are easy to obtain and noninvasive. Therefore, CAS screening using dynamic circulating blood biomarkers is more cost-effective and maybe a complimentary test or a more effective test.

In recent years, miRNA has received widespread attention due to its good stability in circulating body fluids (including whole blood, plasma, serum, and saliva). It can be used as candidate diagnostic and prognostic biomarkers for many diseases and can predict what disease happens. Such as the decrease of miR-637 is a biomarker of poor prognosis in human glioma, which is related to cell proliferation, migration, and invasion [30]. Downregulation of miR-637 increases the risk of pulmonary arterial hypertension by regulating the expression of CDK6 in pulmonary smooth muscle cells [31]. The above studies confirmed the feasibility of miR-637 as a biomarker for disease diagnosis.

Dysregulation of miR-637 is involved in the progression of keloids [32], Osteosarcoma [33], Hirschsprung [34], hepatocellular carcinoma [35]. It is worth noting that miR-637 was marked downregulated in 86 patients with atherosclerosis, which is a putative mechanism of CAS [18]. Downregulation of miR-637 is involved in atherosclerosis and promotes the proliferation and migration of vascular smooth muscle cells by regulating insulin-like growth factor 2 [36]. In patients with essential hypertension, miR-637 is typically reduced [37]. More importantly, miR-637 is reduced in acute ischemic stroke and is negatively correlated with serum procalcitonin [19].

Given the important role of miR-637 in atherosclerosis, hypertension, and acute ischemic stroke, we present some evidence to validate circulating serum miR-637 as a potential diagnostic biomarker for CAS. First of all, there were significant differences in serum miR-637 levels between healthy individuals and CAS patients, and miR-637 showed a downward trend in patients, a pattern of expression consistent with the complications and putative mechanism (atherosclerosis) of CAS. All of the above confirmed the role of miR-637 in the occurrence of CAS. To our knowledge, this is the first study of the expression pattern of serum miR-637 in CAS. According to the degree of carotid artery stenosis, patients were divided into moderate and severe stenosis groups, and the correlation between miR-637 and clinical indicators and degree of stenosis was analyzed. we also found that there are good independent indicators of decreased miR-637 and severe carotid artery stenosis in CAS patients. Subsequently, miR-637 proved to have high potential diagnostic significance in the ROC curve and can distinguish patients from healthy individuals. This is consistent with our original hypothesis.

Since the degree of carotid artery stenosis is a key risk factor for cerebral ischemia events [38]. we previously divided the patients into moderate stenosis group and severe stenosis group according to the degree of carotid artery stenosis for logistics regression analysis, confirming that miR-637 can be used as an independent factor for severe carotid artery stenosis. Moreover, a study has shown that miR-637 is decreased in acute stroke, and is negatively correlated with the expression of serum procalcitonin. It may be that acute ischemic stroke-induced inflammation inhibits miR-637, thus detecting the production and secretion of serum procalcitonin in intestinal neuroendocrine cells in serum, and procalcitonin can be used as a predictive biomarker of adverse survival outcomes in patients [19]. Therefore, we speculated whether miR-637 has a potential predictive ability for cerebral ischemia events. The Kaplan-Meier curve was drawn based on the occurrence of cerebral ischemic events during the 5-year follow-up. Patients with reduced miR-637 levels experienced more cerebral ischemia events, which is the same as the severity of cerebral ischemia events, suggesting that low serum miR-637 levels were a strong predictor of cerebral ischemia events.

There are some limitations to this research. One limitation is that relying solely on phone follow-up may be inadequate to identify cerebrovascular events in a cohort of patients. Secondly, the specificity of miR-637 in our current research may hinder its role as a CAS screening tool, which is another limitation. What’s more, hypertension and dyslipidemia are all risks of CAS. However, there was no significant difference in these indicators between the two groups and a lack of subgroup trade-offs in the expression of other cardiovascular diseases, which may be due to the small size of subjects. Therefore, it is necessary to expand the sample size of subsequent studies and further studies to more carefully define the potential clinical significance of miR-637 in CAS. Finally, the lack of knocking and knockout models and lack of understanding of patient food status is another limitation of studying the potential value of miR-637 to CAS. It is also necessary to carry out this research in the follow-up study.

## Conclusion

In summary, we have demonstrated for the first time that serum miR-637 is in CAS patients is reduced. Decreasing miR-637 is not only a potentially reliable biomarker for the diagnosis of CAS but also a useful indicator for predicting future cerebral ischemic events. In short, this study lays a foundation for the clinical application of miRNA in CAS.
